# Caregiving ability assessment tools for family caregivers of disabled older adults: a scoping review

**DOI:** 10.3389/fpubh.2026.1853102

**Published:** 2026-06-17

**Authors:** Jiahui Fu, Yafang Feng, Xia Si, Cailing Wang, Liyu Zhang, Yinghui Zhang

**Affiliations:** 1 Nursing College, Shanxi Medical University, Taiyuan, China; 2 Department of Nursing, The Second Hospital of Shanxi Medical University, Taiyuan, China

**Keywords:** assessment tools, caregivers, caregiving ability, disabled older adults, scoping review

## Abstract

**Objective:**

To systematically review and analyze the characteristics, psychometric properties, and application status of caregiving ability assessment tools for family caregivers of disabled older adults, providing an evidence-based reference for clinical and community nursing professionals when selecting appropriate instruments.

**Methods:**

Guided by the Arksey and O’Malley framework and the JBI methodology, this scoping review adhered to the PRISMA-ScR guidelines. A systematic search was conducted across nine Chinese and English databases, including CNKI, CBM, VIP, Wanfang Data, PubMed, CINAHL, Web of Science, Cochrane Library, and Embase, with the search timeframe ranging from database inception to October 19, 2025. Two researchers independently screened the literature, extracted data, and summarized relevant information.

**Results:**

We included 25 articles encompassing 21 assessment tools (4 generic and 17 disease-specific). Assessment frameworks have notably shifted from early unidimensional subjective evaluations to multidimensional structures. The most dominant assessed domains include disease-specific knowledge, practical caregiving skills, psychosocial competencies, and resource utilization. While most instruments rely on Likert-type self-reports and demonstrate adequate internal consistency, structural validity and measurement error reporting are frequently limited.

**Conclusion:**

A diverse array of caregiving ability assessment tools exists globally, yet methodological heterogeneity and fragmented disease-specific designs limit broad comparability. Future tool development would benefit from robust theoretical grounding, multi-center longitudinal validation, and modular architectures (e.g., PROMIS-inspired). Implementing tiered assessment strategies can optimally balance evaluation rigor with clinical feasibility, ultimately supporting international healthy aging initiatives and targeted caregiver interventions.

## 1 Introduction

Population aging and the rising prevalence of disability represent profound global public health challenges, driving an unprecedented demand for long-term care services (1, 2). Disabled older adults typically experience significant impairments in daily self-care and health maintenance, resulting in a sustained need for assistance with daily living and professional medical nursing services (3). Globally, and particularly under the cultural context of “filial piety” in China, family caregivers serve as the cornerstone of the long-term care system (4),providing comprehensive home-based care that spans from physical assistance to disease management and emotional support (5, 6). However, assuming this demanding role is challenging, as many family caregivers lack systematic caregiving knowledge and skills (7, 8).

According to the World Health Organization (WHO) (9), healthy ageing emphasizes developing and maintaining the functional ability that enables well-being in older age. When addressing the aging population, it is therefore imperative to broaden the perspective beyond mere physical survival to encompass fundamental components such as quality of life, emotional well-being, and active social participation. Promoting healthy aging requires supporting both the older adults and their family caregivers. Caring for individuals with cognitive or physical impairments is often associated with a progressive decline in the cognitive and physical health of informal caregivers, which may compromise their well-being and their ability to adequately perform caregiving tasks (10, 11). Therefore, it is essential to address caregiver health from a comprehensive socio-healthcare perspective, promoting preventive strategies that minimize emotional risks, such as depression, and physical risks, such as musculoskeletal injuries (12). Furthermore, fostering active living through social networks and physical activities is crucial. For instance, physical exercise, particularly in multicomponent formats, has been shown to improve the psychosocial functioning of the patient-caregiver dyad and reduce caregiver burden, especially when tailored to the family context (13).

To systematically evaluate these gaps, it is essential to clearly conceptualize “caregiving ability”. Farran et al. (14) defined caregiving competence as the purposeful caregiving behaviors undertaken by caregivers based on knowledge, experience, personal habits, and an understanding of the patient’s disease, behavior, and emotions. It encompasses the caregiver’s mastery of objective nursing skills and disease-specific knowledge, their capacity to navigate and utilize social and healthcare resources, and their subjective psychological readiness and resilience in managing caregiving demands. When caregivers exhibit deficits in these core abilities, they face heightened physical and mental exhaustion, financial pressure, and insufficient social support (15, 16), culminating in severe caregiving burden and personal health risks (17). Furthermore, studies indicate that the caregiving ability of family caregivers directly impacts the quality of life and care outcomes for disabled older adults (18). Inadequate caregiving ability not only leads to adverse outcomes—such as falls, pressure ulcers, and unplanned readmissions (19)—but also significantly increases caregivers’ levels of depression and anxiety (20). Therefore, accurately assessing the caregiving ability of family caregivers is of profound clinical significance for identifying specific skill deficits, implementing targeted educational interventions, and ultimately safeguarding the well-being of the caregiving dyad.

Currently, scholars globally have developed various caregiving ability assessment tools tailored for general populations or specific disease types. Nevertheless, existing instruments exhibit significant heterogeneity and methodological limitations regarding the comprehensiveness of assessment dimensions, the rigorousness of psychometric validation, and their cross-cultural adaptability. Specifically, there is a lack of consensus on the core domains of caregiving ability, limited direct comparisons between generic and disease-specific tools, and insufficient cross-cultural validation of these instruments. Based on the scoping review framework proposed by Arksey and O’Malley (21), guided by the Joanna Briggs Institute (JBI) methodology (22), and in adherence with the PRISMA-ScR guidelines, this study systematically collates caregiving ability assessment tools for family caregivers of disabled older adults worldwide. By analyzing the structural characteristics, psychometric properties, and localized application of each tool, this review aims to provide a comprehensive, evidence-based reference for nursing professionals when selecting appropriate assessment instruments for clinical and community practices.

## 2 Materials and methods

### 2.1 Identification of research questions

The conduct and reporting of this scoping review strictly adhered to the Preferred Reporting Items for Systematic reviews and Meta-Analyses extension for Scoping Reviews (PRISMA-ScR) guidelines. The research questions of this study include: ① What caregiving ability assessment tools are currently applied to caregivers of disabled older adults both domestically and internationally? ②What are the characteristics of these assessment tools? ③ What is the application status of these caregiver ability assessment tools in China?

### 2.2 Literature inclusion and exclusion criteria

The Population, Concept, Context (PCC) framework by the Joanna Briggs Institute (JBI) served to establish the eligibility criteria for this scoping review. The JBI methodology was integrated because it provides the most current and rigorous guidance specifically tailored for scoping reviews in evidence-based healthcare, ensuring a comprehensive and transparent approach to identifying diverse assessment tools.

Inclusion criteria: The study population consists of family caregivers of disabled older adult; in this study, disabled older adults are defined as the elderly population suffering from impaired activities of daily living due to aging or diseases such as stroke, dementia, and frailty. The research content involves original articles on the development or application of family caregiver ability assessment tools.

Exclusion criteria: Literature that is repeatedly published or lacks full-text access; studies integrating multiple tools or scales; letters and conference abstracts; and non-Chinese or non-English literature.

### 2.3 Search strategy

A computerized search was conducted across CNKI, CBM, VIP, Wanfang Data, PubMed, CINAHL, Web of Science, Cochrane Library, and Embase. The search timeframe spanned from database inception to October 19, 2025. A combination of Medical Subject Headings (MeSH) and free-text terms was utilized, and the reference lists of the included literature were tracked to supplement the search. Using PubMed as an example, the search strategy was as follows: #1: “Frail Elderly”[Mesh] OR “Activities of Daily Living”[Mesh] OR “Dementia”[Mesh] OR “Alzheimer Disease”[Mesh] OR “Cognitive Dysfunction”[Mesh] OR disability[Title/Abstract] OR disabled[Title/Abstract] OR frail*[Title/Abstract] OR “functional impairment”[Title/Abstract]; #2: “Caregivers”[Mesh] OR “family caregiver*”[Title/Abstract] OR “informal caregiver*”[Title/Abstract] OR “Home Caregiver*”[Title/Abstract]; #3: “Self Efficacy”[Mesh] OR “Clinical Competence”[Mesh] OR “caregiving ability”[Title/Abstract] OR “caregiving capacity”[Title/Abstract] OR competence[Title/Abstract] OR preparedness[Title/Abstract] OR skill[Title/Abstract] OR proficiency[Title/Abstract]; #4: “Surveys and Questionnaires”[Mesh] OR “Psychometrics”[Mesh] OR instrument*[Title/Abstract] OR tool*[Title/Abstract] OR scale*[Title/Abstract] OR measure*[Title/Abstract] OR questionnaire*[Title/Abstract]; #1 AND #2 AND #3 AND #4.

### 2.4 Literature screening and data extraction

The retrieved bibliographic records were imported into EndNote X9 to remove duplicates. Two researchers, who had undergone systematic evidence-based training, conducted the initial screening by reading titles and abstracts based on the inclusion and exclusion criteria. The primary reasons for exclusion at this stage included: study populations not meeting the definition of family caregivers, studies not focusing on caregiving ability assessment, or articles detailing qualitative experiences rather than tool psychometrics. For records meeting the initial criteria, full texts were retrieved and read for a secondary screening. Inter-rater agreement for the study inclusion process was calculated using Cohen’s Kappa coefficient, demonstrating strong agreement (*κ* = 0.85). In case of discrepancies, a discussion was held with a third trained researcher to reach a final consensus. Prior to formal data extraction and COSMIN evaluation, a pilot testing of the extraction form was conducted independently by the reviewers on a random sample of five articles. This pilot phase ensured strict consistency and standardized the reviewers’ understanding of the COSMIN criteria. Data extraction was performed on the included literature, capturing information such as the name of the assessment tool, year of publication, country of origin, assessment content, number of dimensions/items, scoring method, and reliability and validity metrics.

### 2.5 Methodological quality and risk of bias appraisal

The methodological quality and risk of bias of the included caregiving competence and capacity assessment instruments were systematically appraised using the Consensus-based Standards for the selection of health Measurement Instruments (COSMIN) Risk of Bias checklist (23). To ensure a highly objective and rigorous synthesis relevant to the caregiving context, eight core methodological domains (Boxes) were extracted and evaluated: PROM development (Box 1), content validity (Box 2), structural validity (Box 3), internal consistency (Box 4), cross-cultural validity (Box 5), reliability (Box 6), measurement error (Box 7), and hypotheses testing for construct validity (Box 9). Other segments, such as criterion validity (Box 8) and responsiveness (Box 10), were excluded as they were not applicable due to the lack of a clinical “gold standard” or longitudinal intervention designs across the majority of the source studies.

The evaluation strictly followed the COSMIN “worst score counts” principle, meaning that the overall methodological quality rating for each measurement property was determined by the lowest score given to any single item within that specific box. Each domain was graded into four levels: Very Good, Adequate, Doubtful, or Inadequate. For cross-cultural adaptation studies (e.g., Chinese translated scales), focus was placed on Box 5 to ensure linguistic equivalence and Box 6 to check for appropriate statistical indicators (i.e., Intraclass Correlation Coefficient, ICC). Self-designed questionnaires or Delphi-derived expert consensus index systems that lacked empirical large-sample testing were appropriately evaluated based on their documented development properties while acknowledging their statistical validation limits. Two researchers independently performed the quality grading, and any discrepancies were resolved through consensus or consultation with a third methodology expert.

## 3 Results

### 3.1 Literature search results

**Figure 1:**
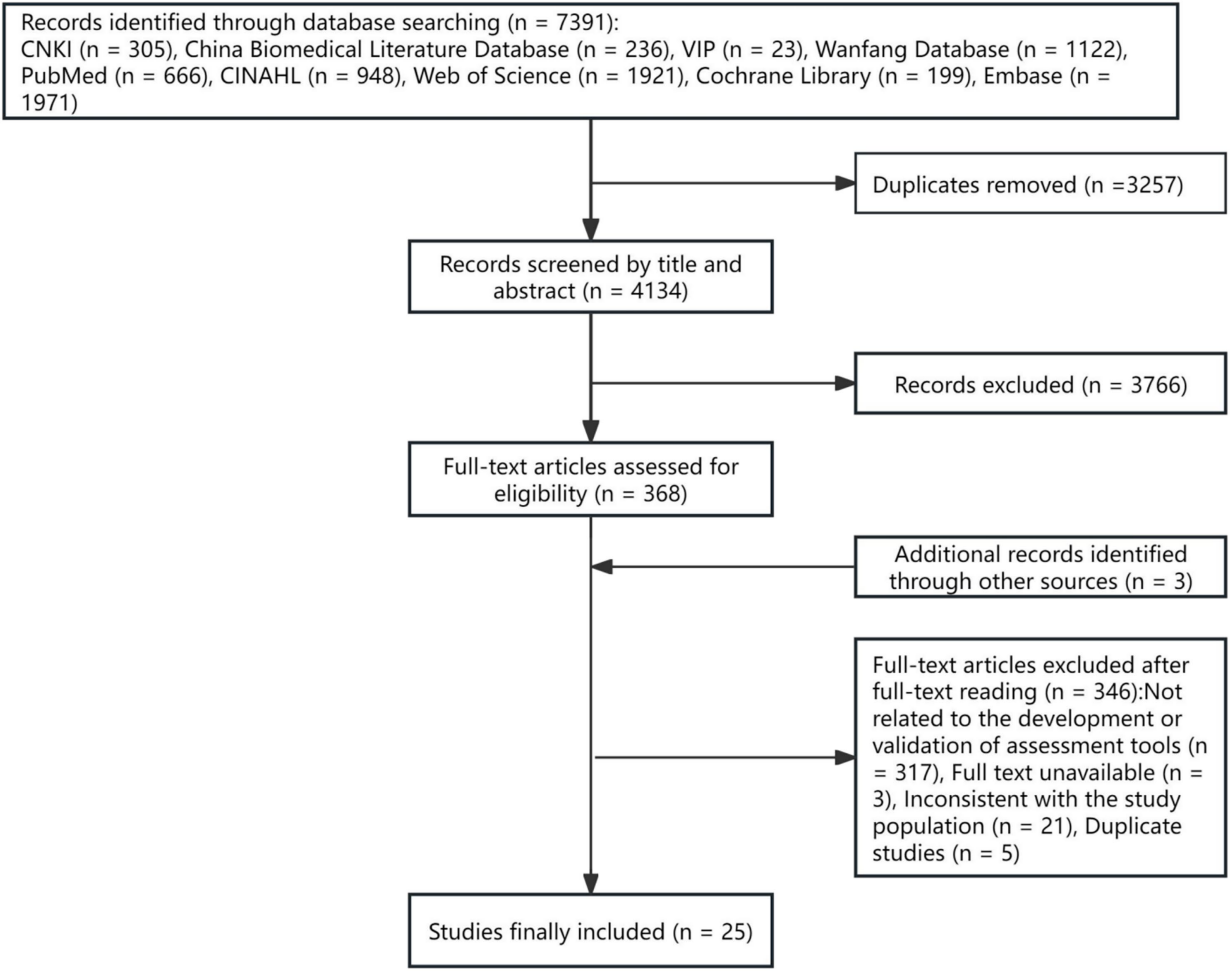
Literature screening flowchart.

An initial search yielded 7,391 relevant articles. After removing 3,257 duplicates using EndNote X9, 4,134 records were screened. Of these, 3,766 records were excluded based on titles and abstracts. A total of 368 full-text articles were assessed, of which 346 were excluded. An additional 3 related articles were identified by tracing references. Ultimately, 25 articles were included in the review. The literature screening process is illustrated in Figure 1.

### 3.2 Basic characteristics of included assessment tools

A total of 21 assessment tools were identified across 25 included studies, comprising 7 tools originally developed outside China and 14 tools either indigenously developed in China or representing validated Chinese adaptations of foreign instruments, with publication years spanning from 1990 to 2025. The basic characteristics of all tools are detailed in Table 1.

**Table 1:** Basic characteristics of the included literature.

1. Tool name	2a. Developing authors (year)	2b. Validation study Author(s)	3. Country/language	4. Evaluation content	5. Scoring method	6. Reliability and validity testing
Caregiver Competency Scale	Pearlin LI et al. (1990) (26)	Cheng H et al. (41) (Chinese version)	U.S./English; Chinese	Single dimension: Perception of nursing work performance, caregiving competence, and confidence	4-point Likert scale	Total Cronbach's α=0.74 (original); Chinese version: I-CVI=0.83-1.00, S-CVI=0.96
Care Preparation Scale	Archbold PG et al. (1990) (27)	—	U.S./English	4 dimensions: Physical care readiness, emotional support readiness, service coordination readiness, stress management readiness	5-point Likert scale (8 core items + 1 open-ended question)	—
Sense of Competence Questionnaire	Vernooij-Dassen MJ et al. (1996) (28)	—	Netherlands/English	3 dimensions: Satisfaction with patient care, satisfaction with caregiver role, personal adverse health outcomes	5-point Likert scale (27 items, total score 27-135)	Dimension Cronbach's α=0.55-0.63
Brief Sense of Competence Questionnaire	Vernooij-Dassen MJ et al. (1999) (29)	—	Netherlands/English	3 dimensions (abbreviated from full SCQ)	5-point Likert scale (7 items, total score 7-35)	Total Cronbach's α=0.76
Caregiving Capacity Questionnaire for Family Caregivers of Disabled Elderly	Li L et al. (2013) (30)	—	China/Chinese	2 dimensions: Disability and relapse prevention knowledge, first aid and rehabilitation skills	5-point Likert scale (10 items, total score 10-50)	Total Cronbach's α=0.844; CVI=0.90; test-retest r=0.924
Dementia Caregiver Knowledge Assessment Scale	Wei Y et al. (2015) (31)	—	China/Chinese	5 dimensions: Personal hygiene, excretion care, dietary care, disease and rehabilitation knowledge, medication safety	Binary scoring (22 items)	Total Cronbach's α=0.626; average CVI=0.95
Informal Caregiver Competence Scale for Elderly Stroke Patients	Araújo O et al. (2015) (32)	Liu M et al. (43) (Chinese version)	Portugal/English; Chinese	Original: 8 dimensions: Nasogastric feeding, personal hygiene, transfer assistance, positioning, technical aid use, toileting, dressing, feeding Chinese version: 7 dimensions: Assistance with meal preparation skills, Nasogastric tube feeding Skills, Assistance with personal hygiene skills, Assistance with transfer skills, Assistance with position adjustment skills, Assistance with toileting skills, Assistance with dressing/dressing-off skills	Original: 4-point Likert scale (32 items); Chinese version: 4-point Likert scale (25 items)	Original: Cronbach's α=0.83, ICC=0.988-0.991; Chinese version: I-CVI=0.875-1.000, S-CVI=0.960, total Cronbach's α=0.833
Chinese Version of Family Caregiving Ability Scale for Elderly Patients	—	Jin X et al. (42)	China/Chinese	3 dimensions: Family caregiving cognitive ability, family cohesion, family support capacity	5-point Likert scale (10 items, total score 10-50)	Total Cronbach's α=0.780; test-retest r=0.665-0.768; split-half reliability=0.745-0.890
Dementia Home Caregiver Competence Questionnaire	Jiang C et al. (2017) (33)	—	China/Chinese	4 dimensions: Dementia-related knowledge, daily home care skills, rehabilitation training skills, caregiver self-stress relief skills	Binary scoring (42 items)	Total Cronbach's α=0.905; CVI=0.90
Dementia Caregiver Ability Assessment Scale	Xie S et al. (2018) (34)	—	China/Chinese	5 dimensions: Disease knowledge, caregiving skills, nursing competence, health maintenance, interpersonal relationships	5-point Likert scale (36 items)	I-CVI=0.857-1.00, S-CVI=0.985; total Cronbach's α=0.936; test-retest r=0.980
Family Caregiver Empowerment Scale for Community-Dwelling Dementia Patients	Sakanashi S et al. (2020) (35)	Xiao H et al. (36) (Chinese version)	Japan/Japanese; Chinese	4 dimensions: Excellent dementia care practice, understanding the nature of dementia care, caring for oneself and the person with dementia, peer support	5-point Likert scale (16 items)	Original: Total Cronbach's α=0.90, dimension α=0.70-0.86; CFI=0.913, RMSEA=0.076: Chinese version: I-CVI=0.850-1.000, S-CVI=0.984; total Cronbach's α=0.904, dimension α=0.661-0.876; test-retest r=0.822; χ^2^/df=2.851, RMSEA=0.096, SRMR=0.066, CFI=0.902
Family Care Capacity Assessment Index System for Community Disabled Elderly	Ren X et al. (2021) (37)	—	China/Chinese	4 primary indicators: Economic capacity, human resources, family cohesion, caregiver competence (12 secondary, 27 tertiary indicators)	Index scoring	Expert authority coefficient=0.88; Kendall's W=0.620-0.647 (P<0.01)
Family Caregiver Care Challenge Scale	Sharif Nia H et al. (2022) (38)	Zhao S et al. (44) (Chinese version)	Iran/English; Chinese	2 dimensions: Effective role-playing challenges, lack of socioeconomic support challenges	5-point Likert scale (10 items, total score 10-50)	Original: Cronbach's α=0.765-0.838, test-retest r=0.900; Chinese version: cumulative variance=64.578%, total Cronbach's α=0.881
Caregiving Skill Deficit Assessment Scale	Cui H et al. (2024) (25)	—	China/Chinese	4 dimensions: Daily life care, safety care, basic disease care, rehabilitation care	Observer rating (89 items, total score 0-135)	Total Cronbach's α=0.883; average CVI=0.998
Older-Adult Caregiving Ability Assessment Scale	Zhou Y et al. (2024) (24)	—	China/Chinese	4 dimensions: Knowledge and skills, care literacy, self-care, seeking support	5-point Likert scale(33 items)	Total Cronbach's α=0.966; S-CVI=0.94; test-retest r=0.84
Caregiving Knowledge and Skills Needs Scale for Family Caregivers of Disabled Elderly	Zhang S et al. (2024) (39)	—	China/Chinese	5 dimensions: Daily living assistance, basic nursing care, rehabilitation nursing care, accident prevention, psychological care	5-point Likert scale (37 items)	Total Cronbach's α=0.862; cumulative variance=73.23%; I-CVI=0.833, S-CVI/UA=0.973
Home Palliative Care Family Caregiver Caregiving Competence Scale	Wang T et al. (2025) (40)	—	China/Chinese	6 dimensions: Care knowledge, general care techniques, special care techniques, care literacy, self-care, resource acquisition	5-point Likert scale (29 items, total score 29-145)	Total Cronbach's α=0.954

The 21 instruments were classified as either generic (*n* = 4) or disease-specific (*n* = 17). The four generic tools—Pearlin’s Caregiver Competency Scale (1990), Archbold’s Care Preparation Scale (1990), Vernooij-Dassen’s Sense of Competence Questionnaire (SCQ, 1996), and its abbreviated version (SSCQ, 1999)—were all developed outside China and are applicable across multiple disease populations. Disease-specific tools predominated, with dementia caregiving representing the most frequently targeted condition (*n* = 8), followed by tools addressing disabled older adults (*n* = 4), general older adult patients (*n* = 2), stroke survivors (*n* = 2), and palliative care (*n* = 1). All tools targeted informal family caregivers, including spouses, adult children, and other relatives providing unpaid care in home or community settings. The majority of tools were designed for home-based or community caregiving contexts, with a smaller subset targeting hospital discharge or clinical settings, and two tools demonstrating applicability across both institutional and community settings.

Considerable heterogeneity was observed across the dimensional structures of included instruments, ranging from unidimensional constructs to frameworks encompassing up to eight domains. Commonly assessed domains included disease-specific knowledge, practical caregiving skills, psychosocial competencies, and resource utilization. A clear developmental trend was evident: early instruments predominantly adopted unidimensional or narrowly framed structures focused on subjective caregiver competence, whereas tools developed post-2013 universally transitioned toward multidimensional frameworks. For example, the Older-Adult Caregiving Ability Assessment Scale (24) incorporates four comprehensive dimensions encompassing knowledge and skills, caregiving literacy, self-care, and support-seeking, reflecting an increasingly holistic conceptualization of caregiving competence that extends beyond task performance to include caregiver wellbeing and resource navigation.

Regarding scoring methods, Likert-type self-report scales constituted the predominant approach (n = 18), with five-point scales most commonly adopted (*n* = 12). Two tools employed binary scoring to assess objective caregiving knowledge, and one tool—the Caregiving Skills Deficit Assessment Scale (25)—uniquely employed an observer-rated methodology requiring trained evaluators to assess skill performance through direct observation and structured demonstration. With respect to theoretical grounding, only six of the 21 tools explicitly grounded their development in established theoretical frameworks, while the majority relied primarily on literature reviews and expert consultation for dimension formulation, reflecting a notable deficit in systematic theoretical grounding across the field.

### 3.3 Psychometric properties

Reliability and validity testing was conducted for 19 of the 21 tools. Overall Cronbach’s *α* coefficients ranged from 0.626 to 0.990, with most total-scale values exceeding the conventional threshold of 0.70. Notably, the dimensional α coefficients of Vernooij-Dassen’s SCQ ranged from 0.55 to 0.63, falling below acceptable thresholds and indicating limited subscale reliability. Test–retest reliability was reported for a subset of instruments, with coefficients ranging from 0.827 to 0.980. Content Validity Index (CVI) values exceeded 0.83 across all tools for which it was reported, and cumulative variance explained by construct validity analyses ranged from 64.578 to 73.23%.

### 3.4 Methodological quality

Methodological quality varied substantially across instruments, as detailed in Table 2. Internal consistency was the strongest-performing domain, with the majority of tools rated Adequate to Very Good, consistent with the near-universal reporting of Cronbach’s *α* values. Content validity was more rigorously documented among Chinese-developed instruments, predominantly established through systematic expert panel consultation and Delphi processes. Structural validity demonstrated the greatest variability, with several earlier instruments and tools relying solely on expert consensus receiving Inadequate or Doubtful ratings.

**Table 2:** Methodological quality of included caregiving competence tools based on the COSMIN risk of bias checklist.

No.	Tool	PROM development	Content validity	Structural validity	Internal consistency	Cross-cultural validity	Reliability	Measurement error	Hypotheses testing for construct validity
Part A:tools developed outside China (*n*=7)									
1	Caregiver Competency Scale (26)	I	D	I	VG	—	N/R	N/R	A
2	Care Preparation Scale (27)	I	N/R	I	VG	—	I	N/R	VG
3	Sense of Competence Questionnaire(28)	I	I	I	VG	—	N/R	N/R	VG
4	Short Sense of Competence Questionnaire(29)	I	I	D	VG	—	N/R	N/R	VG
5	Informal Caregiver Competence Scale for Elderly Stroke Patients (32)	I	VG	D	VG	—	A	N/R	N/R
6	Empowerment Scale for Family Caregivers of Community-dwelling People with Dementia (35)	VG	A	VG	VG	—	N/R	N/R	VG
7	Care Challenge Scale (38)	D	VG	VG	VG	—	A	A	N/R
Part B:tools developed or adapted in China (*n*=14)									
1	Chinese version of the Caregiver Competence Scale (41)	—	I	—	VG	A	I	N/R	VG
2	Caregiving Skills Deficit Assessment Scale (25)	VG	D	I	VG	—	A	N/R	N/R
3	Caregiving Competence Questionnaire (33)	I	N/R	I	VG	—	N/R	N/R	N/R
4	Chinese version of the Family Caregiving Competence Scale (41)	—	I	VG	VG	A	D	N/R	N/R
5	Questionnaire of Caring Ability of Family Caregivers (30)	I	N/R	I	VG	—	N/R	N/R	VG
6	Chinese version of the Abilities' Scale of the Informal Caregiver of the Stroke-dependent Elderly (43)	—	VG	VG	VG	VG	D	N/R	N/R
7	Assessment Index System for Family Caregiving Capacity (37)	VG	N/R	—	—	—	—	—	—
8	Care Competency Scale for Family Caregivers in Home Palliative Care (40)	I	VG	A	VG	—	A	A	N/R
9	Dementia Caregiver Knowledge Assessment Scale (31)	I	VG	VG	VG	—	D	N/R	VG
10	Chinese version of the Empowerment Scale for Family Caregivers of Community-dwelling People with Dementia (36)	—	VG	VG	VG	VG	D	N/R	N/R
11	Caring Ability Assessment Scale for Dementia Caregivers (34)	VG	D	VG	VG	—	D	N/R	N/R
12	Care Knowledge and Skills Demand Scale (39)	VG	D	D	VG	—	N/R	N/R	N/R
13	Chinese version of the Care Challenge Scale (44)	—	VG	VG	VG	VG	A	N/R	VG
14	Older-Adult Caregiving Ability Assessment Scale (24)	VG	VG	VG	VG	—	D	N/R	N/R

VG = Very Good; A = Adequate; D = Doubtful; I = Inadequate; N/R = Not Reported; — = Not Applicable.

The most pervasive methodological gaps were the near-universal absence of test–retest reliability reporting (Not Reported for *n* = 13 tools) and measurement error data (Not Reported for nearly all instruments). Criterion validity and responsiveness data were largely absent across included studies, reflecting the predominance of cross-sectional validation designs in the literature.

## 4 Discussion

### 4.1 Interpretation in light of China’s aging population and socio-cultural realities

This systematic review identified 21 family caregiving ability assessment tools globally (24–44), with a marked increase in tools developed or localized in China post-2013. This proliferation directly reflects the intensifying demand for structured caregiver evaluation driven by China’s rapidly aging population (45, 46) and the national policy shift toward community- and home-based long-term care (47), which has repositioned family caregivers as the primary providers of home-based care within an increasingly formalized care system. Moreover, despite the growing number of available instruments, the practical alignment of these tools with the unique socio-cultural dynamics of Chinese family caregiving—such as filial piety and family-centric care models—remains insufficiently addressed (48, 49). This discrepancy highlights a critical gap that warrants systematic attention as the field moves toward clinical translation in the Chinese cultural context.

### 4.2 Strengths and limitations of generic vs. disease-specific tools

The current landscape of caregiving assessment is divided between generic and disease-specific instruments. Generic tools, pioneered by Pearlin’s Caregiving Competence Scale (26), Archbold’s Preparedness for Caregiving Scale (27), and Vernooij-Dassen’s Sense of Competence Questionnaire (28, 29), offer high versatility and enable broad cross-sectional comparisons of caregiver readiness across diverse disease cohorts (50–54). However, their broad scope often lacks the sensitivity necessary to detect nuanced, condition-specific skill deficits. Conversely, disease-specific assessment tools target distinct conditions or scenarios, enabling a more precise evaluation of unique requisite skills. For instance, dementia care demands proficient skills to manage behavioral and psychological symptoms (55), whereas stroke care predominantly emphasizes rehabilitation training (56). Specific tools, such as the Dementia Home Caregiver Competence Questionnaire (33) integrates specific dementia-related knowledge; and the Informal Caregiver Competence Scale for Elderly Stroke Patients (32, 43) meticulously evaluates practical skills like repositioning, transferring, nasogastric feeding, and utilizing assistive technologies, perfectly aligning with the distinct motor and swallowing dysfunctions prevalent in stroke survivors (57). A deeper critical analysis reveals that the divergence between these tools stems from profoundly different methodological and cultural priorities. Methodologically, generic tools prioritize broad conceptual constructs suitable for evaluating subjective caregiver burden and mutuality. Culturally, many foundational generic tools were developed in Western contexts, emphasizing individual psychological well-being and caregiver identity. In contrast, recently developed or localized disease-specific tools in China frequently emphasize objective, practical task mastery. This shift reflects the pragmatic, urgent demands of home-based care driven by traditional “filial piety,” where family members must rapidly acquire complex nursing skills to substitute for professional institutional care. Despite this localized precision, the rigid boundaries of disease-specific tools restrict their utility, creating a fragmented evaluation environment that fails to capture the comprehensive capability of caregivers when cross-disease challenges arise.

### 4.3 Clinical application status of existing tools

Although the application scope of family caregiving assessment tools is expanding, their practical clinical efficacy requires further empirical validation. Among recognized tools, Archbold’s Preparedness for Caregiving Scale (27) is frequently employed to evaluate caregiving readiness across diverse clinical trials (58). Similarly, Pearlin’s Caregiving Competence Scale (26) is commonly utilized in research involving people living with dementia (59, 60) to measure the efficacy of caregiving interventions and psychological adaptation. Vernooij-Dassen’s SCQ and its short version (28, 29) are predominantly utilized in European contexts (32, 61), serving as foundational assessment metrics for designing targeted caregiver support programs. Comparatively, while indigenous Chinese assessment tools were developed more recently, they have established a solid application foundation in specialized research domains. The Chinese Version of the Family Caregiving Ability Scale for Elderly Patients (42) has been implemented across multiple geographic regions, caregiving settings, and diverse disease populations in China (62–64), demonstrating adequate cultural adaptability. The Caregiving Ability Assessment Scale for Caregivers of People Living with Dementia (34) is applicable to home-based caregivers (51) and is utilized to evaluate the clinical competencies of nursing staff in institutional care facilities (65), indicating broad utility across various research designs (66, 67). Nevertheless, the majority of localized tools remain in preliminary developmental phases, requiring rigorous multi-center, large-sample empirical validations.

### 4.4 Issues of multimorbidity and real-world applicability

In modern geriatric and community care, isolated singular conditions are increasingly the exception rather than the rule. Multimorbidity among disabled older adults is exceptionally common; approximately 32.8% of community-dwelling older adults in China suffer from three or more chronic conditions concurrently (68). Consequently, unidimensional disease-specific tools often fail to capture the comprehensive scope of a caregiver’s actual capabilities (69). When a disabled older adult experiences a clinical collision of cognitive decline and physical functional impairment, a caregiver deemed highly competent by a dementia-specific tool may still critically lack the technical skills required for physical transfers or specialized dietary restrictions. By focusing on a single diagnosis, existing instruments create dangerous assessment blind spots, failing to reflect the complex, overlapping health management needs that family caregivers confront daily in long-term home care environments.

### 4.5 Comparability, usability, and burden for family caregivers

The proliferation of assessment instruments has inadvertently fragmented the research landscape. Within the dementia care domain alone, our review identified nine distinct assessment tools. Although these tools exhibit robust psychometric properties, the significant heterogeneity in their assessment dimensions, item quantities, and scoring systems severely compromises the comparability of results across different clinical and research settings, inadvertently increasing the learning curve and application burden for healthcare professionals. Furthermore, the vast majority of instruments employ self-reported Likert scales, with only one tool utilizing an observer-rating approach. While self-assessments are operationally efficient, they remain highly susceptible to social desirability bias and recall distortion (70), which can significantly compromise measurement accuracy and validity. The Caregiving Skill Deficit Assessment Scale (25) uniquely employs an observer-rating methodology, requiring evaluators to objectively determine skill deficits through direct observation, demonstration, or structured Q&A. However, with an exhaustive 89 items, this scale imposes substantial implementation costs and demands intensive human resources, severely limiting its scalability in routine clinical practice. This inherent conflict between evaluation comprehensiveness and operational efficiency remains a significant hurdle in current clinical applications.

### 4.6 Methodological adequacy: theoretical frameworks and psychometric properties

Early assessment tools primarily concentrated on the subjective experiences of caregivers, frequently equating caregiving ability solely with self-perceived competence. As theoretical paradigms advanced, researchers recognized caregiving ability as a deeply multidimensional construct, encompassing not only subjective perceptions but also objective knowledge, practical operational skills, and the strategic utilization of social resources (71). Post-2013, internationally developed tools have universally transitioned toward multidimensional architectural designs. For example, the Older-Adult Caregiving Ability Assessment Scale (41) incorporates four comprehensive dimensions: knowledge and skills, caregiving literacy, self-care, and seeking support. Notably, our analysis revealed that only a minority of the included tools clearly articulated an underlying theoretical framework: the majority relied primarily on literature reviews and expert consultations for dimension formulation, demonstrating a noticeable deficit in systematic theoretical grounding.

This conceptual gap is directly reflected in the psychometric robustness of the instruments, which subsequently compromises their real-world clinical applicability (72). Our methodological appraisal using the COSMIN Risk of Bias checklist revealed striking heterogeneity. While overall internal consistency is often high, dimensional reliability can be highly problematic (e.g., subscale *α* coefficients between 0.55 and 0.63 in the SCQ) (28).

From a practical standpoint, this poor dimensional reliability implies that healthcare professionals cannot confidently use sub-scores to identify specific skill deficits, potentially misdirecting limited community nursing resources toward the wrong caregiver interventions (73).

Furthermore, crucial evaluations of construct validity are frequently omitted, and there is a near-universal failure to report measurement error and longitudinal responsiveness. In community and home-care settings, the absence of longitudinal responsiveness data is particularly detrimental. It implies that clinicians cannot reliably use these instruments to track a caregiver’s skill acquisition over time (74). Consequently, evaluating the real-world efficacy of tailored caregiver training programs becomes methodologically compromised. If changes in a caregiver’s ability cannot be accurately measured, healthcare systems cannot justify the funding or continuation of essential caregiver support initiatives (75).

More alarmingly, there is a near-universal failure to report measurement error and test–retest reliability. Current psychometric validations predominantly rely on cross-sectional study designs, which inherently lack rigorous assessments of longitudinal validity and observational validity. This highlights a profound clinical limitation: while existing tools may be sufficient for one-time, cross-sectional screening, their lack of responsiveness data makes it impossible to determine if they can accurately detect meaningful, dynamic changes in a caregiver’s skills over time or in response to a training intervention.

### 4.7 Implications for practice and future tool development

In clinical practice, mitigating caregiver burden while maintaining assessment accuracy is paramount. Rather than relying on exhaustive, single-method tools, healthcare systems should adopt a tiered assessment strategy—as recommended by the Family Caregiver Alliance (76) and successfully integrated into comprehensive community programs (6, 77). The caregiver skill-building project developed by Gitlin et al. (78) utilized a comprehensive evaluation strategy that more accurately identified caregiver skill deficits and precise training requirements. By initially screening caregivers with concise, self-reported Likert scales, and subsequently reserving time-intensive, observer-rated evaluations strictly for identified skill deficits, nursing professionals can optimally balance evaluation quality with operational efficiency.

Regarding future tool architecture, developers must aggressively explore hybrid and modular evaluation models to address the complex realities of multimorbidity. Drawing inspiration from the Patient-Reported Outcomes Measurement Information System (PROMIS) (79), future instrument development could profoundly benefit from designing modular assessment tools. These advanced instruments should systematically evaluate universal core competencies required for all disabled older adults, while offering highly flexible, disease-specific modules tailored to individual patient profiles.

Methodologically, future research must elevate its scientific rigor to address the critical gaps identified in this review. First, localized and indigenous tools require extensive deployment across diverse clinical scenarios and demographic populations in China to systematically construct evidence-based application guidelines and enhance their translational value. Second, future instrument development must adhere to COSMIN guidelines by systematically constructing robust theoretical frameworks and utilizing advanced statistical methodologies, such as structural equation modeling, to empirically validate the interactive strengths between dimensions. Most importantly, researchers should prioritize multi-method validity testing, moving beyond isolated cross-sectional snapshots to incorporate multi-center longitudinal cohort data. Establishing test–retest reliability and clinical responsiveness is essential to ensure these tools can dynamically and accurately capture the evolving capabilities of family caregivers across the long-term care continuum.

### 4.8 Limitations

Several limitations of this review warrant acknowledgment. First, the inclusion of only Chinese- and English-language publications may have introduced language bias, potentially excluding relevant instruments developed in other languages. Second, the heterogeneity of included tools in terms of target populations, dimensional structures, and psychometric reporting conventions precluded formal meta-analytic synthesis, limiting the review to descriptive and qualitative comparison. Third, the COSMIN-based appraisal was constrained by incomplete reporting in many primary studies, meaning that ratings of “Not Reported” may reflect reporting deficiencies rather than true methodological absence.

## 5 Conclusion

In summary, this scoping review systematically identified 21 family caregiving ability assessment tools for disabled older adults globally. Although instrument architectures have commendably transitioned from unidimensional subjective evaluations to sophisticated multidimensional frameworks, the current landscape reflects a clear division between broad generic tools and narrow disease-specific instruments. The majority of existing tools do not fully account for the clinical complexities of multimorbidity or the unique socio-cultural dynamics, such as filial piety, inherent in Chinese family structures. Furthermore, methodological appraisals reveal a pervasive reliance on cross-sectional, self-reported designs, with limited data regarding longitudinal responsiveness and systematic theoretical grounding. Overcoming these systemic hurdles through the development of robust theoretical frameworks, modular tool architectures, and tiered clinical assessment strategies will be essential. Ultimately, elevating the methodological rigor and clinical utility of these instruments will better empower family caregivers, optimize home-based long-term care systems, and significantly contribute to the strategic goals of Active Aging, the Healthy China initiative, and global healthy aging frameworks.

## Data Availability

The original contributions presented in the study are included in the article/supplementary material, further inquiries can be directed to the corresponding author.
